# Supplementary figures and images for: Epidemiological, clinical, and laboratory findings for patients of different age groups with confirmed coronavirus disease 2019 (COVID-19) in a hospital in Saudi Arabia
Source: PLoS One. 2021 Apr 29;16(4):e0250955. doi: 10.1371/journal.pone.0250955 (PMC8084156; doi:10.1371/journal.pone.0250955)

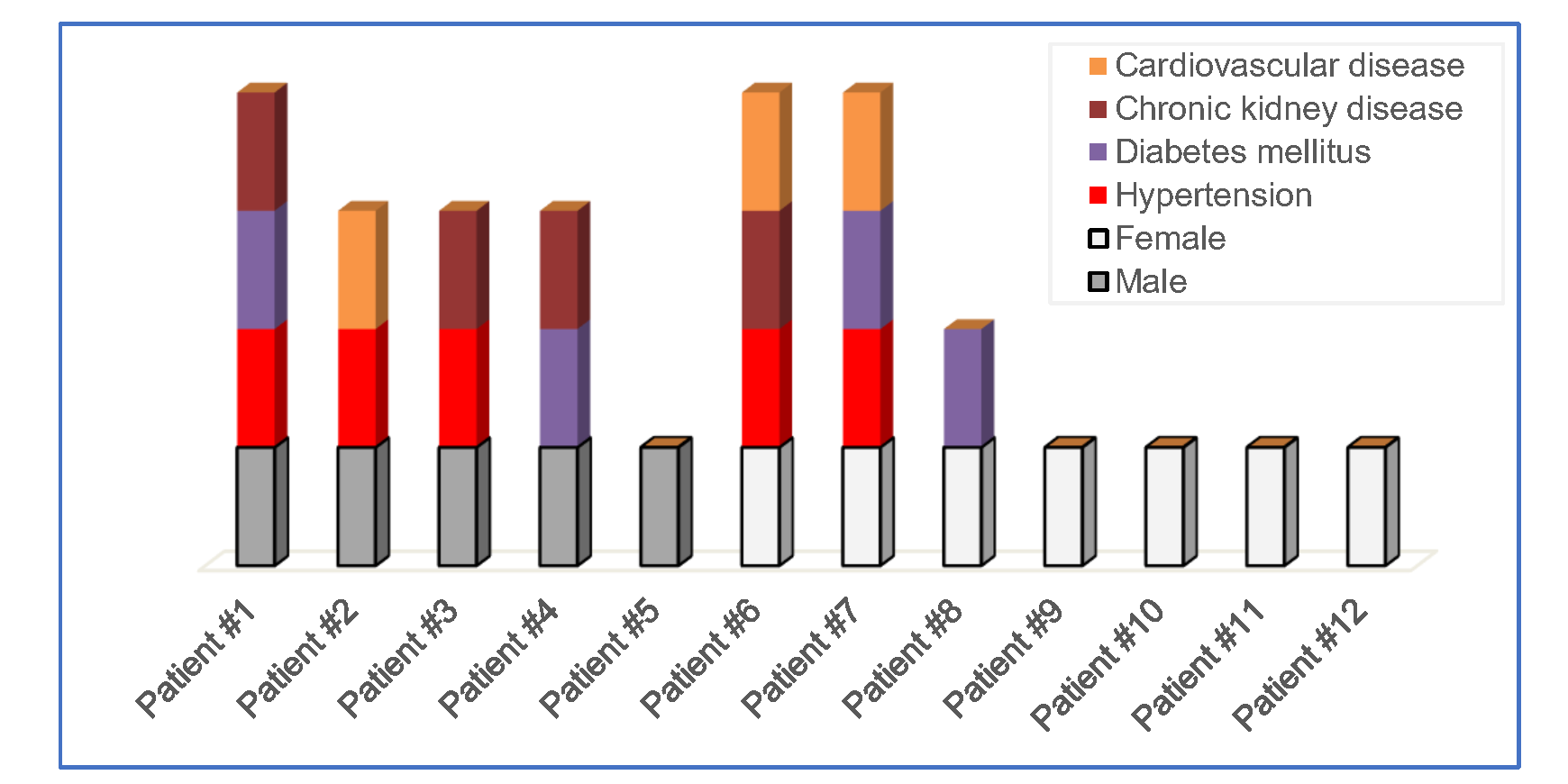

Supplement: S1 Fig — (TIF) [file pone.0250955.s001.tif]
