# Supplementary material for: Epidemiological, clinical, and laboratory findings for patients of different age groups with confirmed coronavirus disease 2019 (COVID-19) in a hospital in Saudi Arabia
Source: PLoS One. 2021 Apr 29;16(4):e0250955. doi: 10.1371/journal.pone.0250955 (PMC8084156; doi:10.1371/journal.pone.0250955)
Supplement: S1 Table — (DOCX) [file pone.0250955.s002.docx]

**S1 Table. Medications and supportive therapies for patients (n=132) with COVID-19 infection**

| **Medication** | **Patient, no (%)** | | |
| --- | --- | --- | --- |
|  | **All patients**  **(n=132)** | **ICU patients**  **(n=33)** | **Dead patients**  **(n=12)** |
| **Antiviral** | 87 (65.9) | 26 (78.8) | 8 (66.7) |
| Favipiravir | 48 (36.6) | 20 (60.6) | 7 (58.3) |
| Oseltamivir | 22 (16.7) | 3 (9.1) | 1 (8.3) |
| Lopinavir/Ritonavir | 17 (12.9) | 3(9.1) | 0 |
|  |  |  |  |
| **Hydroxychloroquine** | 19 (14.4) | 3 (9.1) | 2 (16.7) |
|  |  |  |  |
| **Fluroquinolones** | 81 (61.4) | 23 (69.7) | 8 (66.7) |
| Levofloxacin | 44 (33.3) | 12 (36.7) | 3(25.0) |
| Moxifloxacin | 29 (22.0) | 9 (27.3) | 3 (25.0) |
| Ciprofloxacin | 8 (6.1) | 2 (6.1) | 2 (16.7) |
|  |  |  |  |
| **Macrolide (Azithromycin)** | 50 (37.9) | 9 (27.3) | 3 (25.0) |
|  |  |  |  |
| **Cephalosporins** | 33 (25.0) | 12 (36.4) | 6 (50.0) |
| Ceftriaxone | 24 (18.2) | 8 (24.2) | 4 (33.3) |
| Cefuroxime | 9 (6.8) | 3 (9.1) | 2 (16.7) |
|  |  |  |  |
| **Other antibiotic agents** |  |  |  |
| Amoxicillin/clavulanate | 4 (3.0) | 1 (3.0) | 0.0 |
| Piperacillin/Tazobactam | 6 (4.5) | 4 (12.1) | 1 (8.3) |
| Vancomycin | 7 (5.3) | 4 (12.1) | 3 (25.0) |
|  |  |  |  |
| **Anticoagulant** | 74 (56.1) | 21 (63.6) | 9 (75.0) |
| Enoxaparin | 62 (47.1) | 15 (45.5) | 9 (75.0) |
| Heparin | 7 (5.3) | 2 (6.1) | 0 |
| Apixaban | 5 (3.8) | 4 (12.1) | 0 |
|  |  |  |  |
| **Corticosteroids** | 29 (22.0) | 16 (48.5) | 6 (50.0) |
| Prednisolone | 19 (14.4) | 13 (39.4) | 3 (25.0) |
| Dexamethasone | 10 (7.6) | 3 (9.1) | 3 (25.0) |
|  |  |  |  |
| **Oxygen supplemental** | 34 (25.8) | 20 (60.6) | 8 (66.7) |
| Invasive ventilation | 21 (15.9) | 12 (36.4) | 6 (50.0) |
| Non-invasive ventilation | 13 (9.8) | 8 (24.4) | 2 (16.7) |
